# Flavonoids isolated from the South African weed *Chromolaena odorata* (Asteraceae) have pharmacological activity against uropathogens

**DOI:** 10.1186/s12906-020-03024-0

**Published:** 2020-07-23

**Authors:** Aitebiremen G. Omokhua-Uyi, Muna A. Abdalla, Carmen M. Leonard, Abimbola Aro, Osariyekemwen O. Uyi, Johannes Van Staden, Lyndy J. McGaw

**Affiliations:** 1 Phytomedicine Programme, Department of Paraclinical Sciences, University of Pretoria, Private Bag X04, Onderstepoort, 0110 South Africa; 2 Research Centre for Plant Growth and Development, School of Life Sciences, University of KwaZulu-Natal, Private Bag X01, Scottsville, 3201 South Africa; 3 Deparment of Food Science and Technology, Faculty of Agriculture, University of Khartoum, 13314 Khartoum North, Sudan; 4 Department of Pharmaceutical Sciences, Tshwane University of Technology, Private Bag X680, Pretoria, 0001 South Africa; 5 Department of Animal and Environmental Biology, University of Benin, P.M.B, Benin City, 1154 Nigeria

**Keywords:** South Africa, Compounds, *Chromolaena odorata*, Antimicrobial, Anti-biofilm, Metabolic activity, Anti-adhesion, Toxicity

## Abstract

**Background:**

Urinary tract infections (UTIs) caused by opportunistic pathogens are among the leading health challenges globally. Most available treatment options are failing as a result of antibiotic resistance and adverse effects. Natural sources such as plants may serve as promising alternatives.

**Methods:**

Compounds were isolated from the South African weed *Chromolaena odorata* through column chromatography. Purified compounds were tested for antimicrobial activity using the p-iodonitrotetrazolium chloride (INT) colorimetric method, against uropathogenic *Escherichia coli*, *Staphylococcus aureus*, *Klebsiella pneumoniae*, *Aspergillus fumigatus* and *Cryptococcus neoformans*. Anti-biofilm, anti-adhesion and metabolic inhibition activities were investigated against selected strains. Safety of the compounds was determined against Vero monkey kidney, C3A human liver and colon (Caco2) cells.

**Results:**

Four compounds identified as pectolinaringenin (1), (±)-4′,5,7-trimethoxy flavanone (2), 5-hydroxy-3,7,4′-trimethoxyflavone (3) and 3,5,7-trihydroxy-4′-methoxyflavone) (4) were isolated. Minimum inhibitory concentration (MIC) varied between 0.016 and 0.25 mg/mL. Compounds 2 and 3 showed promising antimicrobial activity against *E. coli*, *S. aureus*, *K. pneumoniae*, *A. fumigatus* and *C. neoformans* with MIC between 0.016 and 0.125 mg/mL, comparable to gentamicin, ciprofloxacin and amphotericin B used as positive controls. Compounds 2 and 3 showed good anti-biofilm and metabolic inhibition activities against *E. coli* and *S. aureus* but weak anti-adhesion activity against the organisms. Low toxicity with selectivity indexes between 1 and 12.625 were recorded with the compounds, indicating that the compounds were rather toxic to the microbial strains and not to the human and animal cells.

**Conclusion:**

Pharmacological activities displayed by compounds 2 and 3 isolated from *C. odorata* and low toxicity recorded credits it as a potential lead for the development of useful prophylactic treatments and anti-infective drugs against UTIs. Although known compounds, this is the first time these compounds have been isolated from the South African weed *C. odorata* and tested for antimicrobial, anti-biofilm, metabolic inhibition and anti-adhesion activities.

## Background

The health challenges posed by infectious diseases caused by opportunistic pathogens globally cannot be overemphasized. Infectious diseases are one of the leading causes of death, especially among the immuno-compromised population [1–3]. Opportunistic pathogens have been reported as a major cause of urinary tract infections (UTIs) in both humans and animals [4]. These include pathogens such as *Escherichia coli*, *Klebsiella pneumoniae*, *Pseudomonas aeruginosa*, *Staphylococcus aureus*, *Enterococcus faecalis*, *Candida albicans*, *Aspergillus fumigatus* and *Cryptococcus neoformans* among others [5–11]. A plethora of antibiotics are used for the treatment of UTIs; however, the development of resistance to most of these antibiotics and adverse side effects have led to a continuous search for alternatives [12, 13].

Opportunistic pathogens contribute to over 80% of opportunistic infections in humans [14]. Biofilms are employed by most opportunistic pathogens as a mechanism of resisting antibiotics in humans [14]. The major factor that promotes biofilm formation is changes in the environment leading to the transition of bacteria from planktonic growth to biofilm formation. This causes changes in the expression of surface molecules, virulence factors, and metabolic status, enabling the bacteria to acquire properties that assist them to survive even in harsh conditions [15–17]. These pathogens can adhere to surfaces such as the lungs, tooth enamel, middle ear, heart valves, and intestinal wall and also to medical devices such as catheters in humans and animals [18]. Apart from the issue of resistance, availability and accessibility of these drugs especially to the rural poor in developing countries is also a barrier to the management of these infections. Hence, interest in the use of plants which are easily reached by the locals as sources of treatments, either as concoctions (herbal remedies) or isolated compounds, against opportunistic infections including UTIs has been growing over the years, as plants contain a wide range of bioactive compounds [19].

*Chromolaena odorata* (L.) King and Robinson, native to the Americas, is an herbaceous perennial flowering shrub belonging to the Asteraceae family [20]. Following its introduction into West Africa in 1937 and to South Africa in 1947 [21–23], the plant has spread to different parts of the continent. Two biotypes are present in Africa, viz. the Asian/West African biotype (AWAB) originating from the Americas and the South African biotype (SAB) originating from Jamaica or Cuba [24–26]. Both biotypes are problematic in their invasive range as they impact negatively on natural vegetation, agriculture and livelihood, causing a decline in biodiversity due to their strong allelopathic properties and ability to survive in harsh conditions, and hence they are regarded as invasive weeds [22, 27, 28].

The AWAB is popularly known in traditional practice for the treatment of skin infections, wounds and inflammation [29–31]. The plant is used in different parts of its invasive range for the treatment of malaria, abdominal and cervical pain, urinary tract infections, gonorrhoea, ulcers, diarrhoea, coughs, colds and skin eruptions [27, 32–37]. A previous study comparing the antimicrobial activity of the two biotypes reported that the SAB contained the same medicinal properties as the AWAB that may be useful in combating antibacterial and antifungal infections [38, 39]. Several bioactive compounds have been isolated from the AWAB including components of essential oils and flavonoids [37]. The isolation of pure compounds responsible for activities possessed by medicinal plants has become an important line of research as a lead or marker for the development of antimicrobial drugs [40]. According to Cos et al. [41], plant products, either as standardised extracts or isolated pure compounds, afford promising opportunities for the discovery of new drugs. This study was designed to isolate compounds from the weed plant *C. odorata* invasive in South Africa and to evaluate them for antimicrobial, anti-biofilm, anti-adhesion and metabolic inhibition activities against selected uropathogenic organisms, as leads for the development of possible treatment against UTIs.

## Methods

### Collection of plant material

The leaves of *C. odorata* were collected from Sappi Cannonbrae plantation, Umkomaas (30^o^ 13′ S, 30^o^ 46′ E) (south coast of KwaZulu-Natal province), South Africa. The management of Sappi Cannonbrae plantation granted us permission to use their site for *C. odorata* research because the plant is an invasive alien plant and a category 1 weed (which must be removed from all properties) in South Africa [39]. A voucher specimen, Coll. No. 5 PRU 123727 *Chromolaena odorata* (L.) King and Robinson*,* was prepared and deposited at the H.G.W.J. Schweickerdt Herbarium, University of Pretoria South Africa, after identification by the herbarium curator Mrs. Elsa van Wyk. Leaves of plants collected were thoroughly cleaned and dried at room temperature in a drying room at the Department of Paraclinical Sciences, University of Pretoria following the guidelines of McGaw and Eloff [42]. The dried leaves were ground to powder using a grinder and stored in sealed glass jars to be used for further study.

### Chemicals, reagents and cell lines

Ethanol, methanol, acetone, acetonitrile, Mueller Hinton broth (MHB), Mueller Hinton agar (MHA), Tryptone soy broth (TSB), Tryptone soy agar (TSA), Sabouraud dextrose broth (SDB), Sabouraud dextrose agar (SDA), Bovine serum albumin (BSA), Phosphate Buffer Saline (PBS), Triton-X 100, p-iodonitrotetrazolium chloride (INT), Thiazolyl Blue Tetrazolium Bromide (MTT), Amphotericin B, ciprofloxacin and Dimethyl sulphoxide (DMSO) were purchased from Sigma Aldrich, South Africa. Gentamicin was purchased from Virbac, New Zealand, Silica gel 60 from Merck, Germany, Minimal essential medium (MEM), Dulbecco’s Modified Eagle’s Medium (DMEM) and foetal calf serum (FCS) from Highveld Biological, South Africa. Cell lines C3A human hepatocyte (ATCC No. CRL-10741) and human colon (Caucasian colon adenocarcinoma (Caco2)) (ATCC HTB 37) were purchased from America Type Culture Collection, while Vero African green Monkey kidney cells was obtained from the collection of the Department of Veterinary Tropical Diseases, University of Pretoria.

### Bulk extraction of plant material

To the powdered leaf material (150 g for methanol and 150 g for acetone), 1500 mL of each solvent were added and placed in the sonicator (EUmax UD200SH) for 1 h. Ice was added to the sonicator to avoid overheating. The mixtures were filtered through Whatman No.1 filter paper using a Buchner funnel. The process was repeated twice and resultant filtrates according to solvent used were combined in a round bottomed flask and concentrated under reduced pressure using a Buchi Rotavapor R-200 (Switzerland) at temperatures between 50 °C and 60 °C. The concentrated extracts were rinsed out of the round bottomed flasks, poured into weighed glass bottles, and placed under a stream of air to dry completely. Dried extracts were weighed with the methanol extract yielding 17.516 g and the acetone extract yielding 16.193 g. Both extracts were mixed together amounting to approximately 34 g. The two solvent extracts (methanol and acetone) were combined owing to the presence of some similar phytoconstituents on developed thin layer chromatographic (TLC) plates under ultraviolet light and sprayed with vanillin reagent when viewed under the ultraviolet light and same retention factor (Rf) values obtained. Also, based on results obtained from preliminary antimicrobial activity screening in a previous study, methanol and acetone were chosen as solvents of choice for bulk extraction to ensure a proper target of active compounds from the plants.

### Isolation of bioactive compounds

Following the method of Suffness and Douros [43] adapted by Eloff [44] with little modification, the combined extract was partitioned between water, hexane, chloroform, ethyl acetate and butanol in this order. Following biological assays against micro-organisms of interest and thin-layer chromatography guided procedures, the hexane (total yield of 11.305 g) and chloroform (total yield of 4.895 g) fractions showing similar antimicrobial results were combined to obtain a crude extract of 16.2 g. This was fractionated through column chromatography using silica gel 60 mixed with hexane to form a slurry in a glass column 10 cm in diameter and 50 cm in length. The mixture was eluted with hexane (hex) followed by stepwise addition of ethyl acetate (EtOAc) (gradient 0 to 100%) followed by ethyl acetate: methanol (MeOH) gradient (till 100% MeOH). For each eluent mixture 1000 mL were used and further decreased to 500 mL. Fractions were collected in round bottomed flasks of 250 mL and concentrated using a rotary evaporator. A total of 76 fractions were collected. Based on TLC monitoring through solvent mobile phases of varying polarity which included benzene/ethanol/ammonium hydroxide (BEA 90:10:1, non-polar/basic); chloroform/ethyl acetate/ formic acid (CEF 5:4:1, intermediate polarity/acidic) and ethyl acetate/methanol/water (EMW 40:5:4.5, polar/neutral), fractions showing similar chromatograms were first combined to obtain 21 fractions. Following a second TLC observation, these fractions were further combined to finally obtain a total of six main fractions: F1 [hex/EtOAc (100:0 and 80:20), 3.7 g], F2 [hex/EtOAc (70:30 and 50:50), 2.04 g], F3 [hex/EtOAc (50:50, 30:70 and 0:100), 1.94 g], F4 [EtOAc/MeOH (90:10 and 70:30), 2.51 g], F5 [EtOAc/MeOH (70:30 and 50:50), 2.1 g], F6 [EtOAc/MeOH (30:70 and 0:100), 2.44 g]. Following biological activity investigation, fraction 2 [F2 [hex/EtOAc (70:30 and 50:50), 2.04 g]], and fraction 3 [F3 [hex/EtOAc (50:50, 30:70 and 0:100), 1.94 g]], were chosen for further purification. Fraction 2 was subjected to column chromatography using a Sephadex LH-20 column with a diameter of 2 cm and length of 50 cm eluted isocratically with methanol and this yielded compound 1 (2.8 mg retention factor = 0.76). Fraction 3 was subjected to the same process and further to preparative thin layer chromatography (PTLC) to yield compounds 2 (67.2 mg, retention factor = 0.89), 3 (67 mg, retention factor = 0.91) and 4 (8.5 mg, retention factor = 0.85).

### Structural elucidation of isolated compounds

The structures of compounds 1, 2, 3, 4 were elucidated using Mass Spectrometry and Nuclear Magnetic Resonance (NMR) spectroscopic techniques. The various compounds were identified through 1-dimensional NMR (^1^H and ^13^C) and 2-dimensional NMR. Data were acquired on a 400 MHz NMR model (Bruker Avance III 400 MHz). Where the quantity of compound was too low a 500 MHz NMR instrument was used. The various compounds were prepared with Ultra Performance Liquid Chromatography (UPLC) grade acetonitrile and water and the mass of each compound was determined with a HR-ESI-MS (Waters Acquity) UPLC system hyphenated to a quadrupole-time-of-flight (QTOF). Chemical shifts were reported with reference to respective deuterated solvent peaks. The structures of compounds isolated were confirmed by comparing their NMR data with published literature.

### Antimicrobial screening

#### Microbial cultures and maintenance

The cultures used were *Escherichia coli* (American Type Culture Collection, ATCC 25922) labelled as 1, *E. coli* isolates (B1989/17 and B1962/17) labelled as 2 and 3 from equine urine, collected from the Department of Veterinary Tropical Diseases, Faculty of Veterinary Science, University of Pretoria, South Africa, *Klebsiella pneumoniae* isolate from commercial chicken eggs [45], *Enterococcus faecalis* (ATCC 29212), *Pseudomonas aeruginosa* (ATCC 27853), *Staphylococcus aureus* (ATCC 29213), *Candida albicans* from a Gouldian finch, *Aspergillus fumigatus* from a chicken and *Cryptococcus neoformans* from a cheetah [45]. These microbial strains were maintained on MHA or SDA for bacteria and fungi respectively, while the isolates were maintained on blood agar.

#### Determination of minimum inhibitory concentration (MIC)

Ten mg/mL of each fraction and 1 mg/mL of isolated compounds were prepared in 10% DMSO and tested against *S. aureus*, *E. coli*, *K. pneumoniae*, *E. faecalis*, *P. aeruginosa, C. albicans, C. neoformans and A. fumigatus*. Cultures were prepared by inoculating each microbial strain from prepared agar plates into sterilized MHB for bacterial and SDB for fungi in sterile McCartney bottles respectively. The inoculums were placed in an orbital shaker incubator. Bacterial strains were incubated at 37 °C for 18 h. Each bacterial culture was diluted in fresh MHB and the absorbance was measured at a wavelength of 560 nm using a microplate reader (Epoch Biotek). The readings were compared to a McFarland standard No. 1 correlating to approximately 3 × 10^8^ cfu/mL). For fungi strains, cultures were incubated at 30 °C for 24 h (*C. albicans)*, 72 h (*C. neoformans)* and 120 h (*A. fumigatus*) respectively. The resultant cells and spores were washed with sterile normal saline and absorbance was measured at a wavelength of 560 nm and turbidity was adjusted to a McFarland standard 1.This was further diluted in a 1:10 ratio in SDB to a turbidity of 5 × 10^5^ cfu/mL. These diluted cultures (bacterial and fungi) were used for the screening following the serial microdilution method of Eloff [46].

### Biofilm inhibition assay

#### Inhibition of planktonic and pre-formed biofilms

A single colony of *E. coli* (ATCC 25922) and *S. aureus* (ATCC 29213), strains were each inoculated into TSB and incubated for 24 h. The biofilm inhibition assay was carried out by adopting the method described by Sandasi et al. [47]. Two stages; planktonic, T0 and biofilm formation, T24 were investigated against the 24 h prepared bacterial cultures. One hundred microliters of each standardised bacterial culture (OD_590_ 1 × 10^6^ cfu/mL) was aliquoted into wells of a sterile flat bottomed 96-well microplate. Plates prepared for 24 h biofilm formation were immediately sealed with an adhesive sealer, placed in the incubator and incubated for 24 h at 37 °C. From the different concentrations of the compounds prepared in their MICs multiplied by 2 (to make final concentrations in the wells to be 0.016, 0.031, 0.063 and 0.125 mg/mL) and a cut-off concentration of 1 mg/mL, 100 μL was added to the wells. Similarly, positive controls (ciprofloxacin and gentamicin with final concentration of 0.01 and 0.032 mg/mL) were included. To the plates prepared for 0 h, the samples were added immediately. Wells containing cultures only, as well as acetone and sterile distilled water used to prepare the samples were included as controls. All plates after the addition of samples were sealed and incubated for 24 h to enable the samples to react with the biofilms.

#### Crystal violet staining assay

The incubated plates were washed with sterile distilled water three to four times after incubation to remove the unattached cells and were placed in the oven at 60 °C for 45 min. To all wells, 100 μL of 1% crystal violet dissolved in distilled water was added and plates were incubated for 15 min at room temperature after which the plates were washed again with sterile distilled water to remove unabsorbed stain. One hundred and twenty-five microliters of 100% ethanol was added to destain the wells. From the wells, 100 μL of the destaining solution was transferred to a new plate and semi-quantitative assessment of biofilm formation was done by measuring absorbance at 590 nm using SoftMax Pro 6 software on a microplate reader (SpectraMax M2). The mean absorbance of the samples was determined, and percentage inhibition was obtained using the equation below:

$$ \mathrm{percentage}\ \mathrm{inhibiton}=\frac{\left(\mathrm{mean}\ \mathrm{of}\ \mathrm{culture}\ \mathrm{control}-\mathrm{mean}\ \mathrm{of}\ \mathrm{sample}\right)}{\mathrm{mean}\ \mathrm{of}\ \mathrm{culture}\ \mathrm{control}}\ \mathrm{x}\ 100 $$

### Metabolic activity inhibition

The ability of the isolated compounds to inhibit the bacterial metabolic activity was tested against *E. coli* and *S. aureus* following the method used by Ramage and Lopez-Ribot [48] with slight modification. The organisms were inoculated in a 96-well microplate following the same biofilm assay procedure described above. The final concentration of each compound in the wells was 0.016 mg/mL, 0.031 mg/mL, 0.063 mg/mL, 0.125 mg/mL and 1 mg/mL respectively. After 24 h of incubation with samples, the plates were washed three times with sterile distilled water and 50 μL of 1 mg/mL INT was added to each well and further incubated in the dark for 2 h. After incubation, absorbance was read at 490 nm using a microplate reader and the percentages of reduction of bacterial metabolic activity in the presence of different concentrations of compounds were calculated using the formula:

$$ \mathrm{percentage}\ \mathrm{inhibiton}=\frac{\left(\mathrm{mean}\ \mathrm{of}\ \mathrm{culture}\ \mathrm{control}-\mathrm{mean}\ \mathrm{of}\ \mathrm{sample}\right)}{\mathrm{mean}\ \mathrm{of}\ \mathrm{culture}\ \mathrm{control}}\ \mathrm{x}\ 100 $$

### Cell adhesion to polystyrene surface

Anti-adhesion activity of isolated compounds was determined using the method of Namasivayam and Vivek [49] with slight modification. To a sterile 96-well flat-bottomed polystyrene microtititre plate, 150 μL of freshly prepared 1% lyophilized powdered globular protein BSA, dissolved in water was added to all wells except for the wells allocated for media only. The plates were incubated for 30 min at 30 °C. Following incubation the wells were washed three times with PBS. To the wells, 50 μL of bacterial cultures equivalent to 1 × 10^6^ cfu/mL read at 590 nm, prepared from overnight cultures were added. This was followed by the addition of isolated compounds 1 mg/mL, ciprofloxacin 0.01 mg/mL and gentamicin 0.032 mg/mL as final concentrations in the wells repectively. The seeded microtitre plates were incubated for 24 h at 37 °C. Non-adhered cells were washed five times with PBS at room temperature. Adhered cells were stained by the addition of 50 μL of 0.1% crystal violet and further incubation for a further 30 min at room temperature. Afterwards, wells were washed with sterile distilled water to remove excess stain followed by the addition of 10 μL of absolute ethanol to fix the adhered cells. Finally, 50 μL of 0.2% Triton X was added to the wells to lyse the cells and the absorbance was read at 570 nm using a microplate reader. Percentage adherence inhibition was calculated using the formula above.

### Cytotoxicity assay

Different concentrations of the compounds ranging from 0.003 to 0.2 mg/mL were tested for cytotoxicity against three cell lines; Vero monkey kidney, C3A and Caco2. Vero cells were grown and maintained in MEM, supplemented with 0.1% gentamicin and 5% foetal calf serum, while Caco2 and C3A cells were grown and maintained in DMEM supplemented with 10% foetal calf serum, 1% non-essential amino acids and 1% penicillin-streptomycin. All cells were incubated in a 5% CO_2_ incubator at 37 °C. Doxorubicin chloride was used as positive control while 10% DMSO used in dissolving the test samples was used as the negative control. The 3-(4,5-dimethylthiazol)-2,5-diphenyl tetrazolium bromide (MTT) assay described by Mosmann [50] with modification by McGaw et al. [51] was used to determine the cytotoxicity of the test samples. The lethal concentration (LC_50_) value was calculated as the concentration of tested sample resulting in a 50% reduction of absorbance compared to untreated cells. Selectivity index (SI) values were calculated against the MIC using the formula:

SI = LC_50_ / MIC.

### Statistical

Microsoft Excel 2016 was used to analyse the data obtained from the different assays.

## Results

### Structural elucidation and identification of isolated compounds

**Fig. 1:**
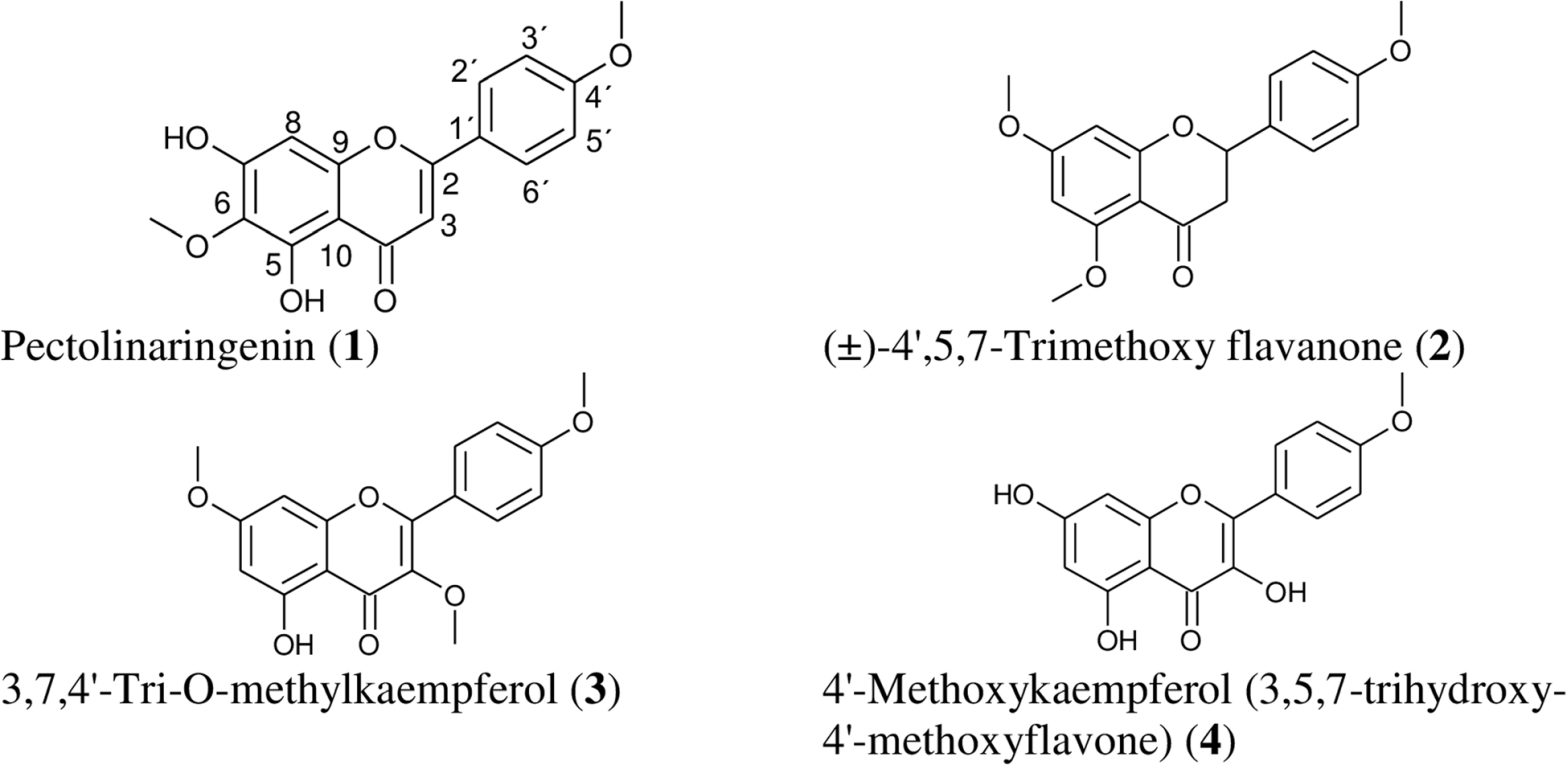
Structures of isolated compounds from South African biotype *Chromolaena odorata*

The structures of isolated compounds were determined after analysing their various NMR spectra and comparison with established literature in the Dictionary of Natural Products and the Chemical Abstracts Services (Scifinder) [52–56] (Fig. 1).

Compound 1 was isolated as a whitish amorphous powder giving a strong UV absorbing band on TLC at 254 nm turning light yellow with vanillin/sulphuric reagent. The retention factor was 0.92 in CEF. The ESIMS delivered a *pseudo*molecular ion peak at 315.0888 [M + H]^+^ and 313.0741 [M-H]^−^ which gave the molecular weight of 314 and the molecular formula C_17_H_14_O_6_ by HRESIMS. The ^1^H NMR showed four aromatic proton signals: each two *ortho*-coupled protons of a *p*-disubstituted benzene appeared at *δ* 8.05 (d, ^3^*J* = 8.6, H-2′,6′) and 7.12 (d, ^3^*J* = 8.6, H-3′,5′) in addition to signals of protons in another rings at *δ* 6.89 (H-3, s) and at *δ* 6.62 (H-8, s). Two methoxy protons appeared at *δ* 3.76 (s, 6-OCH_3_) and *δ* 3.87 (s, 4′-OCH_3_). The ^13^C NMR spectrum revealed 15 carbon signals, nine quaternary carbons at *δ* 182.6 (C-4), 163.7 (C-2), 153.5 (C-5), 131.9 (C-6), 158.1 (C-7), 152.7 (C-9), 104.6 (C-10),123.2 (C-1′), 162.7 (C-4′), and four aromatic carbon signals at δ 103.6 (CH-3), 94.8 (CH-8), 128.8 (CH-2′, and CH-6′), 115.0 (CH-3′, and CH-5′), in addition to two methoxy carbons at *δ* 60.4 (C-6OCH_3_) and 56.1 (C-4’OCH_3_). Additionally, further COSY and HMBC correlations identified the flavonoid structure as pectolinaringenin (5,7-dihydroxy- 4′,6-dimethoxy-flavone) (1) (Fig. 1), by comparing the spectroscopic information with literature data [55].

Compound 2 was isolated as a yellowish-green amorphous powder. On TLC it showed a characteristic colour reaction of a flavonoid compound and retention factor in CEF was 0.91. The mass spectrum showed the *pseudo*molecular peak at m/z 315.1226 [M + H]^+^, consistent with the molecular formula, C_18_H_18_O_5_. The ^1^H NMR revealed a typical flavanone pattern, with protons at positions C-2 and C-3, appeared at *δ* 2.96 (1H, dd, *J* = 2.5, 16.5 Hz, H-3α), 2.68 (1H dd, *J* = 2.5, 16.5 Hz, H-3β), and 5.26 (1H, d, ^3^*J* = 12.6 Hz, H-2). From the ^1^H NMR spectroscopic data, three methoxy protons appeared at *δ* H 3.74 (3H, s, OMe-4′) and 3.80 (3H, s, OMe-5) and 3.73 (3H, s, OMe-7) in addition to four aromatic proton signals at *δ* 6.0 (s, CH-6), 6.06 (s, CH-8), 7.29 (d, ^3^*J* = 8.2, C-2′, and C-6′), and 6.86 (d, ^3^*J* = 8.2, C-3′, and C-5′). ^13^CNMR revealed seven quaternary carbon signals at *δ* 189.4 (C-4), 162.3 (C-5), 165.8 (C-7), 164.9 (C-9), 105.3 (C-10), 123.3 (C-1′), and 160.1 (C-4′), in addition to four aromatic carbon signals at *δ* 93.2 (CH-6), 93.6 (CH-8), 127.8 (CH-2′, and CH-6′), and 114.2 (CH-3′, and C-5′). Moreover, three methoxy carbon signals appeared at *δ* 55.9 (C-4′OMe), 56.1 (C-5OMe) and 55.6 (C-7OMe) in addition to oxygenated methine at *δ* 78.9 (CH-2) and a methylene carbon signal at 45.3 (CH_2_–3). Further COSY and HMBC correlations and a search in Chemical Abstracts Services (Scifinder) confirmed the structure as (±)-4′,5,7-trimethoxy flavanone (Fig. 1).

Compound 3 was isolated as yellow crystals showing a strong UV absorbing band on TLC at 254 nm and turned to brown with vanillin/sulphuric acid reagent and retention factor was 0.852. The ESIMS afforded a *pseudo*molecular ion peak at 329 [M + H]^+^, which gave the molecular weight 328 and the molecular formula C_18_H_16_O_6_ by HRESIMS. Compound 3 was closely related to compound 4 as the ^1^HNMR spectrum showed a typical flavonol pattern. For instance, a methoxy signal detected at *δ* 3.75 (3H, s, OMe-3) in addition to two methoxy proton signals at *δ* 3.77 (3H, s, OMe-4′) and 3.80 (3H, s, OMe7). Moreover, two aromatic proton signals appeared at *δ* 6.45 (d, ^3^*J* = 2.1, CH-6) and 6.49 (d, ^3^*J* = 2.1, CH-8) in addition to two aromatic proton signals of a *p*-disubstituted benzene observed at *δ* 7.97 (d, ^3^*J* = 8.9, H-2′,6′) and 6.90 (d, ^3^*J* = 8.9, H-3′,5′). ^13^C NMR spectrum afforded nine quaternary carbon signals at *δ* 156.9 (C-2), 138.3 (C-3), 182.9 (C-4), 158.9 (C-5), 164.3 (C-7), 158.3 (C-9), 105.9 (C-10),123.0 (C-1′), 162.2 (C-4′), four aromatic carbon signals at *δ* 93.5 (CH-6), 91.1 (CH-8), 130.2 (CH-2′, and CH-6′), and 114.6 (CH-3′, and C-5′) and the methoxy carbon signals at *δ* 60.1 (C-4′OMe), 55.5 (C-3OMe) and 55.9 (C-7OMe). COSY as well as HMBC correlationsidentified compound 3 as 3,7,4′-tri-O-methylkaempferol (5-hydroxy-3,7,4′-trimethoxyflavone) (Fig. 1).

Compound 4 was isolated as a yellow amorphous powder, which gave a strong UV absorbing band on TLC at 254 nm and turned to yellowish-brown with vanillin/sulphuric acid reagent. The retention factor when eluted with CEF was 0.89. The ESIMS afforded a *pseudo*molecular ion peak at m/z 301.0710 [M + H]^+^, which gave the molecular weight 300 and the molecular formula C_16_H_12_O_6_ by HRESI MS. The ^1^H NMR spectrum showed the typical pattern of a flavonoid: four aromatic proton signals were observed at *δ* 8.10 (H-2′, 6′), 6.97 (H-3′, 5′), 6.39 (H-8) and 6.23 (H-6). Additionally, one methoxy signal appeared at *δ* 3.81 (4′-OCH_3_). The ^13^C NMR spectrum revealed nine quaternary carbons (Cq) at *δ* 181.6 (C-4), 161.8 (C-7), 161.2 (C-4′), 161.1 (C-5), 157.6 (C-9), 146.0 (C-2), 137.7 (C-3), 123.3 (C-1′) and 104.4 (C-10), in addition to four aromatic methine carbon signals at 129.3 (CH-2′, 6′), 114.2 (CH-3′, 5′), 98.8 (CH-6) and 94.2 (CH-8) and a methoxy singlet at 55.9 (4′-OCH_3_). 1D and 2D NMR data obtained for compound 4 showed typical signals for flavonol especially the 3-hydroxyflavone at *δ* 137.7 (C-3) as well as correlations confirming the flavonol skeleton. This compound was identified as 4′-methoxykaempferol (3,5,7-trihydroxy-4′-methoxyflavone). A literature search in the Dictionary of Natural Products and the Chemical Abstracts Services (Scifinder) afforded the compound 4′-methoxykaempferol, of which the spectral data was comparable to literature (as described in Mabry & Harborne [52] (Fig. 1).

### Antimicrobial activity of fractions and isolated compounds

Fractions and subfractions subjected to antimicrobial screening showed varying MIC values (from 0.08 to 2.5 mg/mL) (Tables 1 and 2). The hexane and chloroform fractions had the best activity against most of the strains tested with MICs of 0.08 and 0.16 mg/mL. The antimicrobial activities of isolated flavonoid compounds are presented in Table 3. Compounds 2 and 3 inhibited all bacterial strains tested while compound 1 inhibited only *S. aureus*, *K. pneumoniae* and *E. coli* ATTC strain and the Compound 4 only *S. aureus* and *E. coli* ATCC strain. Compound 2 exhibited very good antibacterial activity against *E. coli* ATCC and isolates and *S. aureus* (MIC = 0.031 mg/mL). Activity against *S. aureus* was better than that of the positive control ciprofloxacin, a very promising result. The average mean of the compound was 0.05 ± 0.04, almost comparable to that of gentamicin which had average MIC of 0.06 ± 0.11. Antibacterial activity exhibited by compound 3 (MIC = 0.016 mg/mL) against *S. aureus* was better than that of ciprofloxacin with MIC = 0.063 mg/mL. Amongst all isolated compounds tested against the two fungal isolates, very good antifungal activity was displayed by Compound 2 with MIC = 0.016 mg/mL against *C. neoformans* and *A. fumigatus* at 48 h, and the compound had the same average MIC value of 0.07 ± 0.06 as amphotericin B. Compound 3 showed MIC = 0.031 mg/mL only against *A. fumigatus* at 48 h, comparable to amphotericin B used as the positive control.

**Table 1:** Minimum inhibitory concentration of fractions against selected microbial strains in mg/mL

Organism	Time (h)	Hexane	Chloroform	Butanol	ETAC	Water	Gent	Cipro	Amp B
*E. coli* (1)	**–**	0.08*	**0.16**	0.63	1.25	0.63	**0.02**	**0.002**	NA
*K. pneumoniae*	**–**	**0.16**	**0.16**	0.63	1.25	0.63	0.500	0.050	NA
*E. faecalis*	–	0.63	**0.16**	0.63	> 2.5	0.63	0.500	ND	NA
*P. aeruginosa*	–	0.63	0.31	0.63	1.25	0.63	**0.008**	ND	NA
*S. aureus*	**–**	**0.16**	**0.16**	0.63	1.25	**0.16**	**0.008**	0.063	NA
*C. albicans*	24	0.08*	0.63	0.63	1.25	0.63	**NA**	**NA**	**0.031**
	48	0.31	2.5	> 2.5	> 2.5	> 2.5	NA	NA	0.063
*A. fumigatus*	48	0.31	2.5	1.25	> 2.5	1.25	NA	NA	**0.031**
	72	0.31	2.5	> 2.5	> 2.5	> 2.5	NA	NA	0.063
	120	> 2.5	> 2.5	> 2.5	> 2.5	> 2.5	NA	NA	0.125
*C. neoformans*	48	0.63	2.5	1.25	2.5	2.5	NA	NA	**0.008**
	72	0.63	2.5	1.25	> 2.5	> 2.5	NA	NA	0.125
	120	> 2.5	> 2.5	> 2.5	> 2.5	> 2.5	NA	NA	0.125

*ND* not detected, *NA* not available, *ETAC* ethyl acetate, *Gent* gentamicin, *Cipro* ciprofloxacin, *Amp B* amphotericin B

Value with asterisk are regarded as those fraction(s) with good activity

**Table 2:** Minimum inhibitory concentrations of combined fractions obtained from silica gel column against most susceptible strains in mg/mL

Organism	Time (h)	Sub-Fraction					
		F1	F2	F3	F4	F5	F6
*S*. *aureus*	–	> 2.5	0.08*	**0.16**	**0.16**	0.3	0.31
*K*. *pneumoniae*	–	1.25	0.3	**0.16**	0.63	0.313	**0.16**
*E*. *coli* (1)	–	0.63	**0.16**	**0.16**	0.63	0.63	0.63
*E*. *faecalis*	–	1.25	0.31	0.31	2.5	2.5	0.63
*C. albicans*	48	2.5	> 2.5	2.5	2.5	2.5	2.5
	72	> 2.5	> 2.5	**>** 2.5	> 2.5	> 2.5	> 2.5
*A*. *fumigatus*	48	1.25	**0.16**	0.08*	0.63	1.25	0.31
	72	> 2.5	0.63	0.63	> 2.5	> 2.5	> 2.5
	120	> 2.5	> 2.5	> 2.5	> 2.5	> 2.5	> 2.5
*C*. *neoformans*	48	> 2.5	0.31	0.31	0.31	**0.16**	0.31
	72	> 2.5	0.63	1.25	2.5	2.5	> 2.5

*F1* fraction 1, *F2* fraction 2, *F3* fraction 3, *F4* fraction 4, *F5* fraction 5, *F6* fraction 6. Values with bold indicate promising antimicrobial activity while value in asterisk indicate best activity

**Table 3:** Antimicrobial activity of isolated flavonoid compounds in mg/mL

Organism	Time (h)	Compounds						
		1	2	3	4	Gent	Cipro	Amp B
*S. aureus*	–	0.25	**0.031**	**0.016**	0.25	0.008	0.063	NA
E. coli (1)	–	0.25	**0.031**	**0.063**	0.125	0.016	0.002	NA
*E. coli* (2)	–	> 0.25	**0.031**	0.25	> 0.25	0.031	5 × 10^−5^	NA
*E. coli* (3)	–	> 0.25	**0.031**	0.25	> 0.25	0.008	1 × 10^−3^	NA
*K. pneumoniae*	–	0.25	0.125	0.125	> 0.25	0.25	0.05	NA
Mean	–	0.25	0.05	0.14	ND	0.06	0.02	NA
SD	–	0	0.04	0.11	ND	0.11	0.03	NA
*C. neoformans*	48	> 0.25	**0.016**	> 0.25	> 0.25	NA	NA	0.08
	72	> 0.25	0.125	> 0.25	> 0.25	NA	NA	0.125
*A. fumigatus*	48	0.063	**0.016**	**0.031**	0.25	NA	NA	0.031
	72	0.25	0.125	0.25	> 0.25	NA	NA	0.063
	120	> 2.5	> 2.5	> 2.5	> 2.5	> 2.5	NA	NA
Mean		ND	0.07	ND	ND	ND	NA	0.07
SD		ND	0.06	ND	ND	ND	NA	0.04

*E. coli* (1): *E. coli* (ATTC25922) reference strain; *E. coli* (2): *E. coli* (B1989/17) isolate; *E. coli* (3); *E. coli* (B1962/17) isolate; Gent = gentamicin; Cipro = ciprofloxacin; Amp B = amphotericin B; 1 = compound 1; 2 = compound 2; 3 = compound 3; 4 = compound 4; NA: not applicable; ND: not detected; SD: standard deviation. Number in bold indicates good antimicrobial activity

### Biofilm inhibition activity

**Fig. 2:**
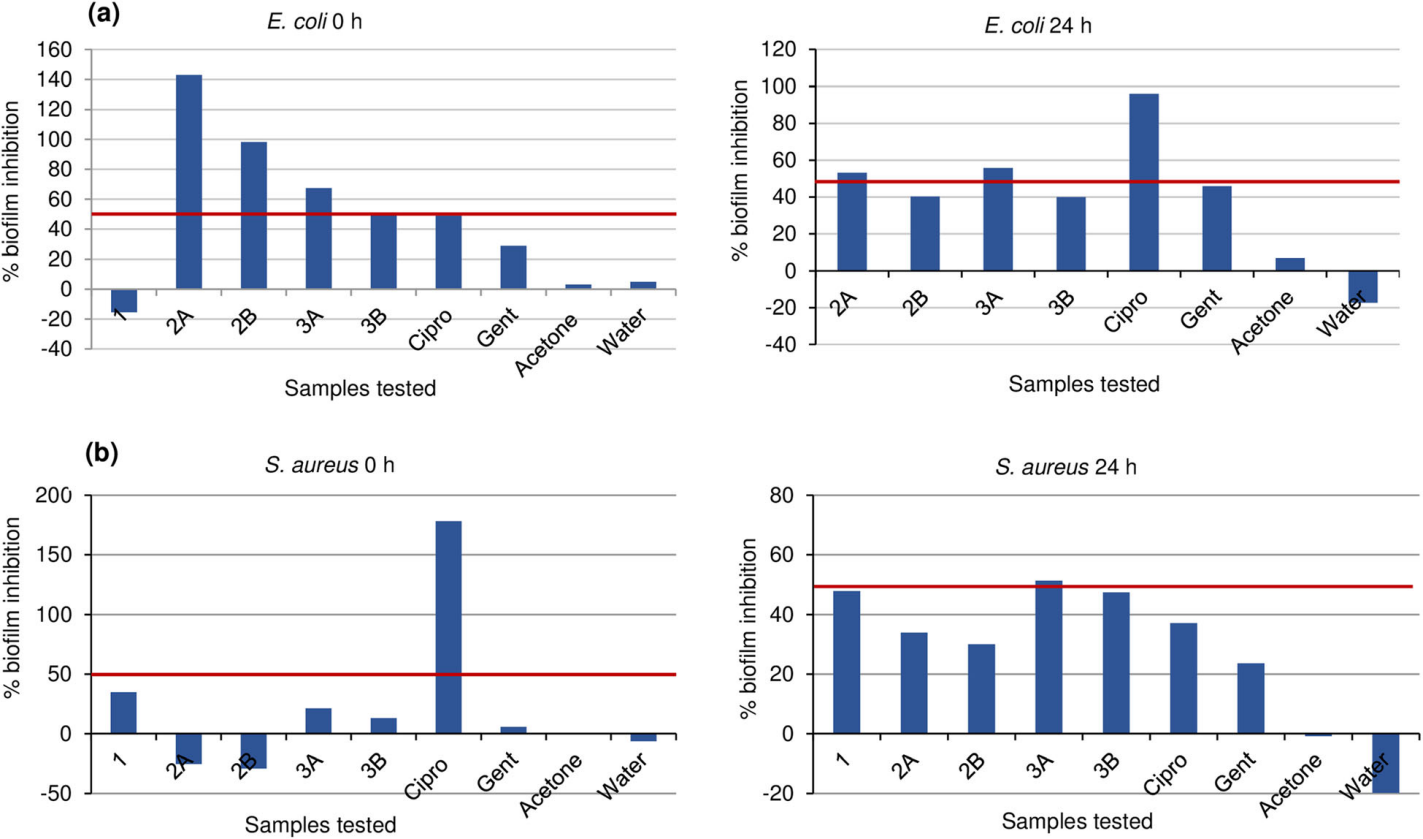
(**a**-**b**) Biofilm inhibition of *E. coli* and *S. aureus* at 0 and 24 h of isolated compounds 1, 2 and 3. 1: compound 1 at 1 mg/mL; 2A: compound 2 at 1 mg/mL; 2B: compound 2 at 0.063 mg/mL MIC against *E. coli* and *S. aureus*; 3A: compound 3 at 1 mg/mL; 3B: compound 3 at 0.125 mg/mL against *E. coli* and 0.032 mg/mL against *S. aureus*

Results obtained with the isolated flavonoids at the planktonic and biofilm formation stages against *E. coli* and *S. aureus* are shown in Fig. 2 (a-b). Among the three compounds tested, only 2 and 3 had anti-biofilm properties with good activity against *E. coli*, but for *S. aureus* poor inhibition was observed except for only 3 which had activity higher than 50% at 24 h.

### Metabolic activity inhibition

**Fig. 3:**
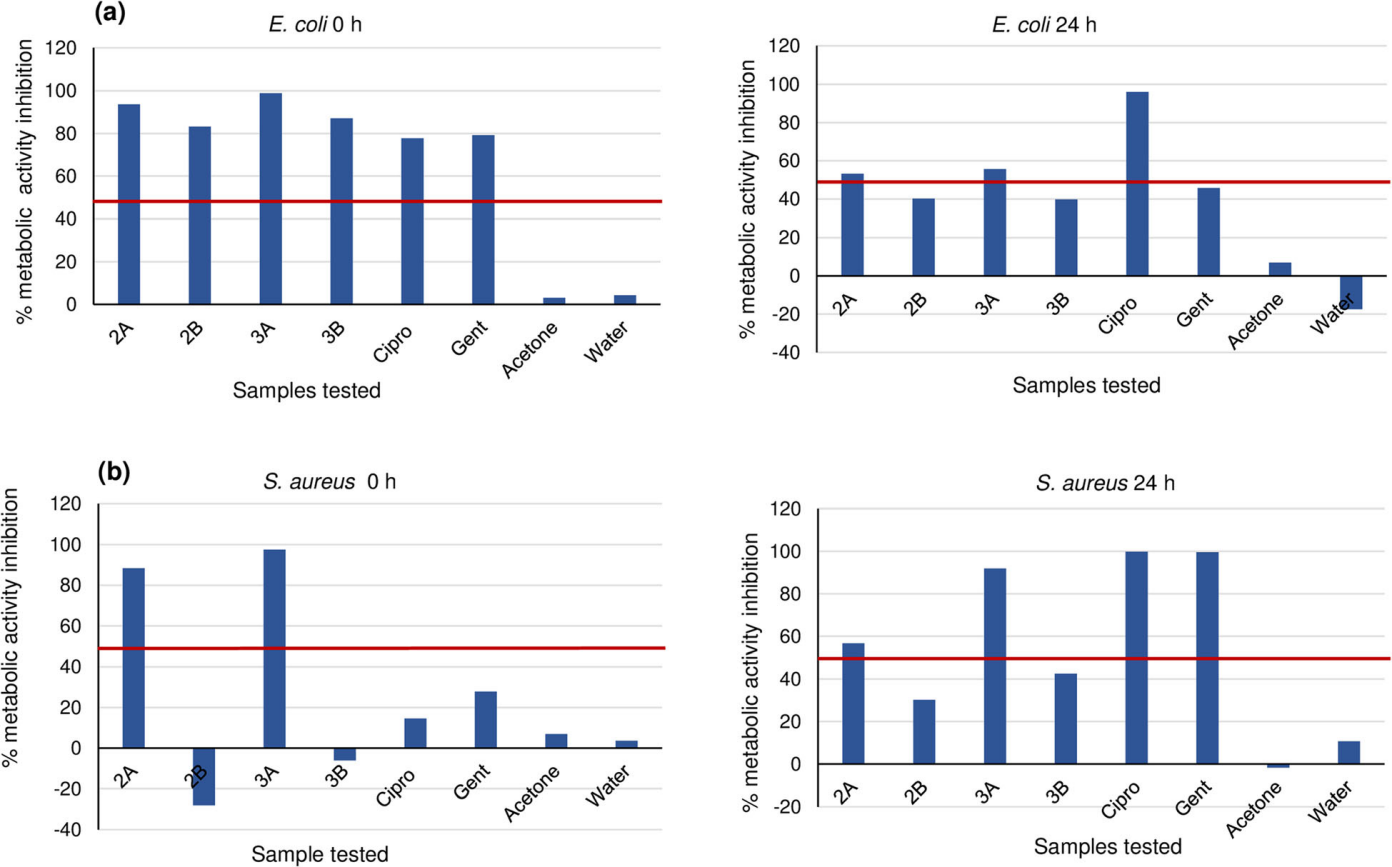
(**a-b**) Metabolic activity inhibition of *E. coli* and *S. aureus* at 0 and 24 h by compound 2 and 3 compared with ciprofloxacin and gentamicin. 2A: compound 2 at 1 mg/mL; 2B: compound 2 at 0.063 mg/mL MIC against *E. coli* and *S. aureus*; 3A: compound 3 at 1 mg/mL; 3B: compound 3 at 0.125 mg/mL against *E. coli* and 0.032 mg/mL against *S. aureus*

Results of the metabolic activity inhibition of *E. coli* and *S. aureus* by the flavonoid compounds are presented in Fig. 3(a-b). Metabolic activity inhibition between 18 and 99% at 0 h and 24 h against *E. coli* and *S. aureus* was observed with compounds showing weak to good inhibitory activity at both 1 mg/mL concentration and at MIC levels.

### Inhibitory effect of bacterial adhesion to polystyrene surface

**Fig. 4:**
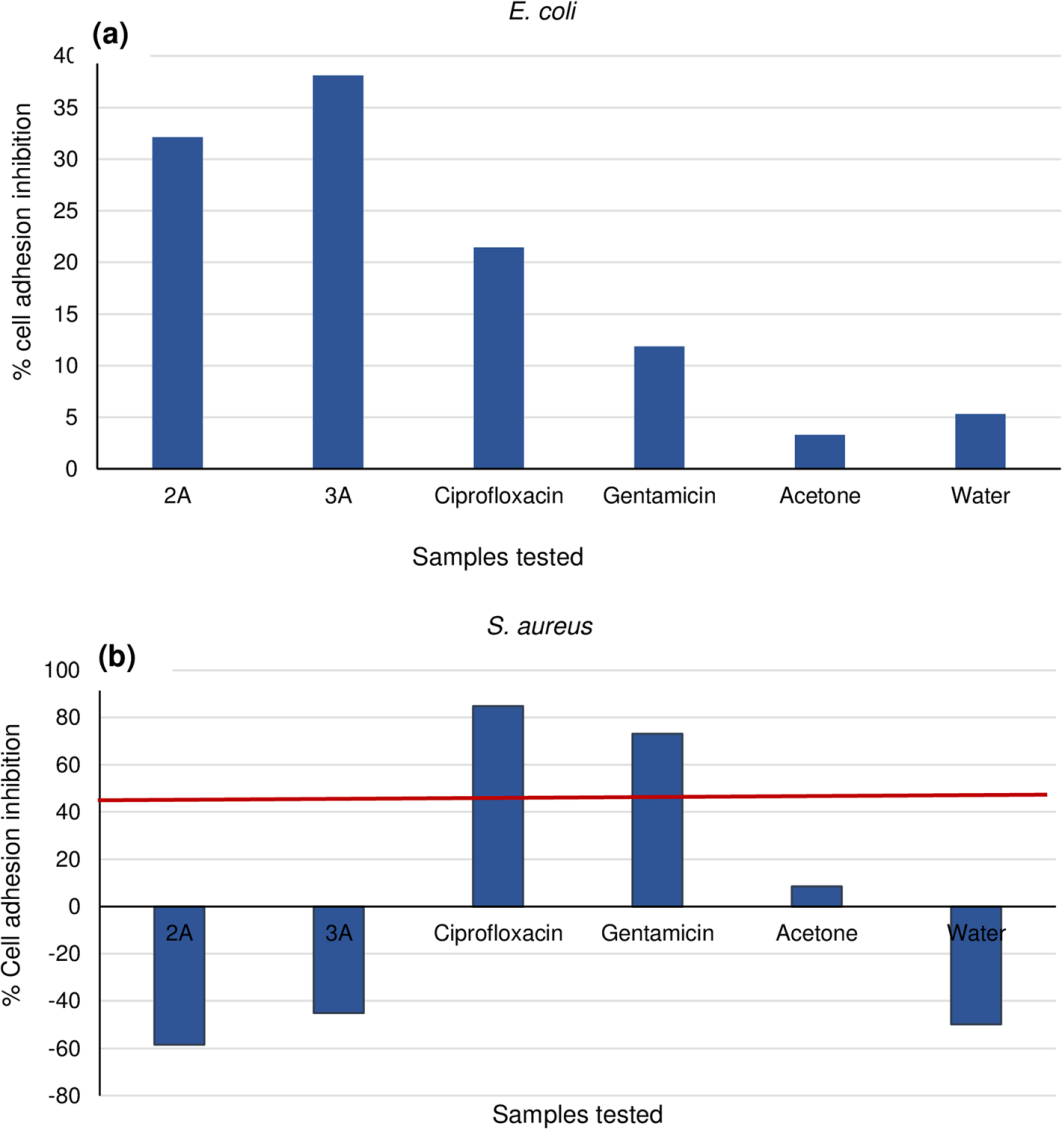
(**a-b**). Adherence inhibition of *E. coli* and *S. aureus* by isolated compounds 2 and 3 at 1 mg/mL concentration compared to positive controls

Figure 4(a-b) shows the inhibitory activity of isolated compounds compared to standard antibiotics against *E. coli* and *S. aureus* in a 96-well polystyrene microplate. Although both compounds showed some inhibitory effect, activity was weak against *E. coli* while against *S. aureus* the compounds tended to support bacterial adhesion.

### Cytotoxicity screening

Table 4 shows the result of the cytotoxicity levels of the screened flavonoid compounds against Vero Monkey kidney, Caco2 and C3A cells. None of the isolated compounds was toxic against the cells at the highest concentration (0.2 mg/mL) tested. Table 5 represent the selectivity index (SI) of the compounds against the bacterial and fungal strains tested. The SI values calculated indicate that the antimicrobial, anti-biofilm and metabolic inhibition activities of the compounds were not because of toxicity of the compounds to the microbial strains.

**Table 4:** Lethal concentration of compounds, and controls tested against Vero monkey kidney, Caco2 and C3A cell lines in mg/mL

Sample	Vero cells	Caco2 cells	C3A cells
1	NA	NA	NA
2	0.200	> 0.200	> 200
3	0.197	0.159	> 200
4	> 0.200	0.193	NA
Ciprofloxacin	> 0.200	> 0.200	NA
Gentamicin	> 0.200	> 0.200	NA
Doxorubicin	0.0028 ± 0.012	0.0004	0.0033

1 = compound 1; 2 = compound 2; 3 = compound 3; 4 = compound 4; *NA* not available. > 0.200 indicates that no sign of toxicity was noticed at the highest concentration tested

**Table 5:** Selectivity index of microbial strains tested against Vero and Caco2 cell lines

Microbial strain	Cell line	Time (h)	Compound 2	Compound 3	Compound 4
		Selectivity index (LC_50_/MIC)			
*S*. *aureus*	Vero	–	6.516	12.313	ND
	Caco2	–	ND	9.938	0.772
*E*. *coli* (1)	Vero	–	6.515	3.127	ND
	Caco2	–	ND	2.52	1.544
*E*. *coli* (2)	Vero	–	6.452	0.788	ND
	Caco2	–	ND	0.636	ND
*E*. *coli* (3)	Vero	–	6.452	0.788	ND
	Caco2	–	ND	0.636	ND
*K*. *pneumoniae*	Vero	–	1.616	1.576	ND
	Caco2	–	ND	1.272	NA
*C. neoformans*	Vero	48	12.625	NA	ND
		72	1.616	NA	ND
	Caco2	48	ND	NA	NA
		72	ND	NA	NA
*A. fumigatus*	Vero	48	**12.625**	6.355	ND
		72	1.616	0.788	ND
	Caco2	48	ND	5.129	0.7772
		72	ND	0.636	NA

*ND* not detected due to LC_50_ greater than the highest concentration of 0.2 mg/mL tested showing no toxicity, *NA* not available as a result of the sample not being able to inhibit the tested fungal strain at highest concentration

## Discussion

### Isolated compounds

Five fractions from *C. odorata* (hexane, chloroform, butanol, ethyl acetate and water) tested against five bacterial and three fungal strains exhibited antimicrobial activity from weak to good activity. However, the hexane and chloroform fractions showed better activity against most of the strains especially *E. coli*, *S. aureus* and *K. pneumoniae* which were used for the bio-guided isolation in this study. Most of the sub-fractions collected were more active against *E. coli*, *S. aureus*, *K. pneumoniae*, *A. fumigatus* and *C. neoformans* (Table 2) which finally resulted in isolation of four compounds with Rf values of 0.85, 0.89, 0.91 and 0.92 respectively. Characterization and structural elucidation of the compounds revealed pectolinaringenin (5,7-dihydroxy-4′,6-dimethoxy-flavone) (1), (±)-4′,5,7-trimethoxy flavanone (2) 3,7,4′-tri-O-methylkaempferol (5-hydroxy-3,7,4′-trimethoxyflavone) (3) and 4′-methoxykaempferol (3,5,7-trihydroxy-4′-methoxyflavone) (4).

The presence of the compound 1 in *C. odorata* agrees with that of Wollenweber et al. [57] and Raman et al. [58] who also isolated this compound from the widespread AWAB *C. odorata,* which has been reported as a very useful medicinal plant in its invasive range. Compound 1 seems to be one of the major flavonoids present in the sunflower Asteraceae family as it has been isolated from other plants of the family which include; *Schkuria pinnata* (Lam*)*, *Buccharis uncinella* DC [59]., *Cirsium japonicum* DC [60]. etc. The compound has also been isolated from other plants belonging to other families, e.g. *Plumeria ambigens* S. F and *Plumeria floribunda* L. (Apocynaceae) [61], *Clerodendrum phlomidis* L.f. (Verbenaceae) [55], *Kickxia ramosissima* (Wall.) (Plantaginaceae) [62], and *Lippia* species (Verbenaceae) [63]. The compound has also been obtained through hydrolysis of the rutinose unit of pectolinarin isolated from *Linaria reflexa* L. (Plantaginaceae) [64]. Compound 2 has been isolated from *Launaea arborescens* (Batt.) Murb*.* (Asteraceae) [65]. Compound 3 has also been isolated from *Dodonaea viscosa* Jacq. var. *angustifolia* (L.f) Benth. (Sapindaceae). Compound 4 is commercially sold with the name Diosmetin, and is a common compound found in citrus fruits [66]. It has also been isolated from other plant species which include *Chrysanthemum morifolium* Ramat (Asteraceae) [67], *Premna odorata* Blanco (Lamiaceae) [68] and *Avena fatua* L. (Poaceae) [69].

### Antimicrobial activity of isolated compounds

The antimicrobial activity of the isolated compounds from *C. odorata* against *S. aureus*, *E. coli*, *K. pneumoniae*, *A. fumigatus* and *C. neoformans* (Table 3) showed that the plant is a promising source of antimicrobials, especially the compounds 2 and 3. The antibacterial activity displayed by these two compounds may be attributed to the presence of the O-methoxy group present at carbon-4 and carbon-7 located in the A and B rings of the two compounds as illustrated in Fig. 1. Better antifungal activity displayed by 2 compared to 3 against *C. neoformans* and *A. fumigatus* may be due to the O-methoxy group located at carbon-5 in the A ring whereas that of 3 is located in carbon-3 of the C ring. In a study carried out by Teffo et al. [70] where compound 3 was investigated against *S. aureus* and *E. coli*, no activity was recorded, contrary to the present finding where MIC values of 0.063 mg/mL against *E. coli* and 0.016 mg/mL against *S. aureus* were observed. However, their findings affirm the result of this study for compound 4 activity against *S. aureus* and *E. coli* where the same MIC values of 0.125 and 0.25 mg/mL were observed.

Compound 1 showed weak antimicrobial activity in this study except against *A. fumigatus* where good activity was observed at 48 h. The poor activity exhibited may be influenced by the two methoxy rings at carbon-4 and carbon-6. Derivatives of this compound may be a more effective antimicrobial. The compound has been reported to display antiprotozoal activity against *Plasmodium falciparium* and *Trypanosoma cruzi* [62], and antioxidant and anti-inflammatory activities [62, 71]. Other biological activity has been reported on this compound, for example, the compound isolated from a Thai traditional herbal formulation (Benchalokawichian) has been reported to demonstrate anti-allergic activity of the skin [72]. The presence of this compound in *C. odorata* may explain why the AWAB is said to be effective against skin infections [30, 31, 73]. The compound has demonstrated hepatoprotective activity in a rat model of hepatic injury induced by D-galactosamine [71]. It has also been reported to display antiproliferation activity by inducing apoptosis and deregulation of B-cell lymphoma 2 (Bcl-2) expressions in MCF-7 breast cancer cells [60]. It has shown antitumor activity in mice with S180 and H22 tumor cells [69]. The compound has also been reported to display larvicidal activity against *Aedes aegyptia*, *Anopheles stephensi* and *Culex quinquefasciatus* and larvicidal pulpicidal and oviposition deterrent activities against *Helicoverpa armigera* [55, 74, 75]. The compound inhibited constitutive and interleukin-6-induced STAT3 signaling and diminished the accumulation of STAT3 in the nucleus, blocking STAT3 DNA-binding activity in osteosarcoma cells [76]. This may further explain why AWAB is used in traditional medicine for the treatment of malaria, as a mosquito repellent and as an insecticide [77–81].

Although compound 4 had weak antimicrobial activity against the microorganisms tested, the compound has been reported to be very useful as a nutritional and dietary supplement because of its ability to increase testosterone levels in the blood, assist in the proper functioning of the heart and the circulatory system, lower cholesterol levels in the blood, increase anabolic processes and help in burning fat [82]. It has also been reported to possess good antioxidant activity [70].

### Biofilm, metabolic activity and cell adhesion inhibition

Among the isolated compounds tested, only 2 and 3 showed good inhibitory activity against the planktonic and mature biofilms of *E. coli* and the mature biofilm of *S. aureus* even at the MIC levels tested (Fig. 2a-b). This indicates that these compounds have preventive ability against infection caused by these organisms and may also serve as effective treatment. The same trend was observed with the compounds 2 and 3 against the metabolic activity of *E. coli* and *S. aureus* both at the planktonic and biofilm stages (Fig. 3a-b), except for *S. aureus* where only the MIC of 0.063 mg/mL did not show inhibition at the planktonic stage. The ability of these compounds to inhibit the metabolic activity of the bacterial strains implies that these compounds may have the ability to distort the metabolic activity of the organisms, although a further study is recommended to establish this. In comparing activity displayed between the Gram-positive and Gram-negative strains, better anti-biofilm activity was observed by the compounds against *E. coli*. The trend of anti-biofilm and metabolic inhibitory activities exhibited by these compounds against *E. coli* and *S. aureus* in this study agrees with that of Mohsenipour and Hassanshahian [83] where they tested *Thymus vulgaris* L*.* (Lamiaceae) extracts against both strains.

Anti-adherence activity displayed by the compounds against *E. coli* was weak, while for *S. aureus* the isolated compounds promoted bacterial adhesion to the protein BSA on the polystyrene surface. It may not be impossible that the poor anti-adhesion obserevd in this study may be due to the method used, hence, further studies is suggested. Athough good anti-adhehence activity was not noticed with this plant in this study, some other plants of the Asteraceae family have been reported to have good anti-adherence activity [84].

### Safety evaluation through cytotoxicity

The cytotoxicity screening of the flavonoid compounds showed that none of the compounds or positive controls was toxic against Vero monkey kidney, Caco2 or C3A human cells at the highest concentration tested. This implies that the pharmacological activity displayed against the bacterial and fungal strains was not because of toxicity. Selectivity index values between 1 and 12 for all compounds tested indicate that the compounds were more toxic to the bacterial and fungal strains than the cells investigated. This implies that the isolated compounds may be useful in the treatment of UTIs caused by the investigated strains, without much fear of safety [8, 85, 86]. However, in vivo studies will be relevant with regards to this.

## Conclusions

The global health challenges caused by UTIs resulting from microbial infections cannot be overlooked, as they are among the major contributors to high morbidity and mortality, especially in immuno-compromised individuals. Following a biological assay-guided isolation, four compounds were isolated from the SAB biotype of *C. odorata*. Amongst the four compounds isolated from the alien invasive plant*,* only two compounds, (±)-4′,5,7-trimethoxy flavanone and 5-hydroxy-3,7,4′-trimethoxyflavone showed promising antimicrobial activity against *E. coli*, *S. aureus*, *K. pneumoniae*, *A. fumigatus* and *C. neoformans,* which are implicated as some of the major pathogens causing UTIs. The results from this study show that the investigated flavonoid compounds may be useful in the fight against UTIs. The compounds also showed promising anti-biofilm activity against *E. coli* and *S. aureus.* This supports their potential development into useful prophylactic treatments and as anti-infective drugs. Also, their ability to inhibit the metabolic activity of the bacterial strains implies that these compounds may have the ability to distort the metabolic activity of the organisms, although a further study is recommended to establish this. This is the first time these compounds have been isolated from this biotype of *C. odorata*. This study is the first to investigate the antimicrobial, anti-biofilm, metabolic activity and cell adhesion inhibition potentials of the isolated compounds from the SAB *C. odorata.* A further study to evaluate the compounds as possible adjuvant treatments with conventional antibiotics will be appropriate.

## Data Availability

Dataset and materials can be made available upon reasonable request from the senior author.

## Abbreviations

UTIs: Urinary tract infections
MHB: Mueller Hinton broth
MHA: Mueller Hinton agar
TSB: Tryptone soy broth
TSA: Tryptone soy agar
SDB: Sabouraud dextrose broth
SDA: Sabouraud dextrose agar
BSA: Bovine serum albumin
PBS: Phosphate Buffer Saline
INT: p-iodonitrotetrazolium chloride
MTT: Thiazolyl Blue Tetrazolium Bromide
DMSO: Dimethyl
FCS: foetal calf serum
SAB: South African Biotype *Chromolaena odorata*
AWAB: Asian/West African Biotype *Chromolaena odorata*
